# Editorial: Nutritional epigenetics and cancer prevention: mechanisms and biomarkers

**DOI:** 10.3389/fnut.2026.1823817

**Published:** 2026-04-20

**Authors:** Ferenc Budán, Duygu Ağagündüz, Zoltán Gyöngyi, Bence Raposa, Dávid Szép

**Affiliations:** 1 Medical School, Institute of Physiology, University of Pécs, Pécs, Hungary; 2 Faculty of Health Sciences Nutrition, Dietetics Department, Gazi University, Ankara, Türkiye; 3 Medical School, Institute of Public Health, University of Pécs, Pécs, Hungary; 4 Faculty of Health Science, Institute of Basics of Health Sciences, Midwifery and Health Visiting, University of Pécs, Pécs, Hungary

**Keywords:** chemical carcinogens, chemoprevention, environmental pollution, epigenetics, nutrition science

## 1 Introduction

The global incidence of cancer has risen steadily in recent decades and is expected to increase further due to population aging. Approximately 44% of cancer-related deaths are considered preventable. Combinational strategies involving bioactive compounds may exert synergistic effects through epigenetic modifications, including changes in DNA methylation, non-coding RNA expression, and histone modifications (1).

Advances in genomic and epigenetic technologies now allow more precise identification of individual susceptibility, supporting targeted prevention strategies (2). Understanding the molecular mechanisms of carcinogenesis enables the identification of predictive biomarkers and contributes to improved prevention (3).

Pharmaceuticals combined with life-style changes, nutritional factors or phytonutrients, and immune-based therapies are promising. Their efficacy may be influenced by epigenetic regulation, potentially modulated by diet. Additional factors—such as nutrigenomics, microbiome interactions, physical activity, and metabolic or inflammatory diseases—also warrant attention and are closely linked to dietary habits (4).

Molecular modeling, along with *in vitro* and *in vivo* approaches, are capable to elucidate molecular pathways and biomarkers. Clinical and epidemiological studies, especially when supported by artificial intelligence, may reveal complex associations between lifestyle and health outcomes. Reviews and meta-analyses also play a key role in synthesizing evidence (5). Ultimately, such knowledge may guide targeted interventions to improve life expectancy through modification of lifestyle factors, particularly diet (6).

## 2 Summary of contributing articles

Tian et al. in their article *Methionine restriction in cancer: a dietary insight for therapy* reviewed methionine restriction (MR) as an anti-cancer strategy. Methionine, an essential amino acid, is critical for protein synthesis and metabolic processes. Many cancer cells depend on external methionine due to defects in its recycling pathway, making MR a promising therapeutic approach. MR disrupts one-carbon metabolism and reduces levels of S-adenosylmethionine, a key methyl donor, thereby affecting DNA and histone methylation. Experimental studies show that MR suppresses tumor growth and enhances the efficacy of conventional therapies, highlighting its potential in precision oncology.

Ogunlakin et al. in their research article: *Ethnomedicinal validation of Telfairia occidentalis L. leaf: a dual experimental and computational approach to uterine leiomyoma therapy* investigated the effects of *Telfairia occidentalis* leaf extract on monosodium glutamate-induced uterine fibroids in rats. The plant, traditionally used in Nigerian medicine, showed moderate antioxidant activity and dose-dependent inhibition of key enzymes. Treatment reduced hormone levels and improved uterine and ovarian tissue structure. Phytochemical analysis identified compounds such as beta-carotene and lutein. These findings suggest antifibrotic effects through antioxidant, enzymatic, and hormonal mechanisms, supporting its traditional use.

Ige et al. elucidated in their research article: *Antioxidant and ameliorative effects of selected Nigerian plants on hormonal imbalance associated with dysmenorrhea in albino rats* the antioxidant and hormonal effects of several Nigerian medicinal plants used to treat dysmenorrhea. Non-steroidal anti-inflammatory drugs are commonly used, but due to their side effects safer alternatives are warranted. The studied plants contained bioactive compounds such as flavonoids and phenolics. Antioxidant assays showed varying activity among the plants, and *in vivo* experiments demonstrated increased estradiol levels. Overall, several species showed strong antioxidant and hormone-modulating effects, supporting their potential for plant-based therapies.

Ye et al. established in their research article *Prognostic impact of nutritional indictors based on Lasso-Cox regression for non-muscle-invasive bladder cancer* a prognostic model for non-muscle invasive bladder cancer patients undergoing Bacillus Calmette-Guérin therapy. Using Lasso-Cox regression, the study identified blood-based biomarkers associated with recurrence, treatment failure, and disease progression. Newly developed nutritional risk indices demonstrated strong predictive value and improved risk stratification compared to existing models. These findings suggest that nutritional biomarkers can support personalized treatment decisions, although further validation is warranted.

## 3 Conclusions

The studies in this Research Topic highlight the strong connections between nutrition, metabolism, and disease-related biological processes. They elucidate dietary interventions such as methionine restriction, the therapeutic potential of plant-derived compounds, and the role of nutritional biomarkers in predicting clinical outcomes.

Despite differences in methodology, all studies indicate that metabolic processes, oxidative balance, and nutritional status significantly influence disease development, progression, and treatment response. These findings highlight the growing integration of nutrition science, molecular biology, and clinical research in understanding complex diseases.

## 4 Future research directions

Further research should validate experimental findings on dietary interventions and plant-derived compounds through well-designed clinical studies. The integration of nutritional and metabolic biomarkers into clinical models represents a promising direction for improving risk stratification and treatment decisions.

Advances in metabolomics, molecular epidemiology, and systems biology may further clarify how nutritional factors influence disease mechanisms. In the long term, such interdisciplinary approaches may enable the development of personalized preventive and therapeutic strategies in which nutrition plays a central clinical role.
